# Immune Microenvironment in Colorectal Cancer: A New Hallmark to Change Old Paradigms

**DOI:** 10.1155/2011/174149

**Published:** 2011-11-24

**Authors:** Luis de la Cruz-Merino, Fernando Henao Carrasco, David Vicente Baz, Esteban Nogales Fernández, Juan José Reina Zoilo, Manuel Codes Manuel de Villena, Enrique Grande Pulido

**Affiliations:** ^1^Clinical Oncology Department, Hospital Universitario Virgen Macarena, Avenida Dr. Fedriani s/n, 41009 Sevilla, Spain; ^2^Clinical Oncology Department, Hospital Universitario Ramón y Cajal, Carretera de Colmenar Viejo, Km 9,100, 28034 Madrid, Spain

## Abstract

Impact of immune microenvironment in prognosis of solid tumors has been extensively studied in the last few years. Specifically in colorectal carcinoma, increased knowledge of the immune events around these tumors and their relation with clinical outcomes have led to consider immune microenvironment as one of the most important prognostic factors in this disease. In this review we will summarize and update the current knowledge with respect to this intriguing and complex new hallmark of cancer, paying special attention to infiltration by T-infiltrating lymphocytes and their subtypes in colorectal cancer, as well as its eventual clinical translation in terms of long-term prognosis. Finally, we suggest some possible investigational approaches based on combinatorial strategies to trigger and boost immune reaction against tumor cells.

## 1. Introduction

The term immunity derives from the Latin word “*immunitas*”, referred to the exemption of Roman senators in legal procedures while holding their public office. In time, this term has won many other meanings; in the Medical field it is employed to describe the reaction of an organism towards the aggression caused by external pathogens, initially infectious agents. More recently, antigens derived from neoplastic processes have been reported as responsible for triggering immune responses. Most solid tumors induce an immune response in the host, confirmed by histopathological studies. In this sense, tissue affected by colorectal cancer is invaded by immune cells from the host, suggesting that the amount of lymphocytes may play a prognostic role with a potential impact upon patient's survival [1].

In Europe, 376.000 new cases of colorectal cancer are diagnosed each year, with mortality close to 203.700 patients. It is one of the most frequent cancers worldwide, in both genders [2, 3], and in most developed countries; as a result of screening and diagnostic techniques and advances in the field of surgery and radio-chemotherapy, survival has significantly increased in the last decades. Most tumors affecting the colorectal area are adenocarcinoma-like which in most cases are well or moderately differentiated. If colorectal neoplasias invade through the muscularis mucosa into the submucosa, local host reactions take place in cancer tissue and proinflammatory cells accumulate along the margins of the tumor, creating an immune microenvironment and triggering an immune response targeted towards the tumor [4].

## 2. Tumor Immune Microenvironment: Immune-Surveillance and Tumor-Infiltrating Lymphocytes (TILs)

In normal conditions, the immune system is an effective “gate-keeper” against cancer. Antitumor activity of the immune system is initially mediated by innate immunity, mainly with effector cells such as Natural Killer (NK) cells, neutrophils, and macrophages. Subsequently, adaptive immunity mechanisms are activated. This response is specific and generates memory cells, mainly B and T-cells which encompass the humoral and cellular immunity [1].

Cancer development can be explained, at least in part, by the success of the immunosuppressive escape mechanisms displayed by the tumor against the host's immune response [5]. This scenario is an area of great interest in the research of tumor microenvironment, with evidence supporting the hypothesis that a potent and effective immune reaction against certain tumor antigens (epitopes) may overcome escape mechanisms, leading to the elimination and control of the cancer [5]. The aforementioned evidence led to Hanahan and Weinberg, among others, to postulate recently avoidance of immune-surveillance as a new hallmark of cancer [6]. In this sense, cancer cells may escape the innate and immune host responses mainly by two mechanisms: selection of non-immunogenic tumor cell variants (immunoselection) or by active suppression of the immune response (immunosubversion) [5, 6].

Tumor-infiltrating lymphocytes (TILs) are located in the inflammatory infiltrates in tumor islets and in the peritumoral stroma of solid tumors [7]. TILs include cytotoxic T-lymphocytes (CD8), NK cells, and helper T-lymphocytes (CD4). Among the latter, there is a subpopulation of cells known as regulatory T-cells (Tregs), formerly suppressor T-cells, main actors in suppressing and controlling the immune response [8]. Whereas Treg cells carry out a physiological role in the prevention of autoimmune events in the host to avoid a disproportionate response to self antigens, in the case of malignant neoplasias their presence seems more related to immunosuppressive mechanisms preventing immunomediated tumor destruction [9]. The relationship between CD8/NK and Treg cells in the tumor-peritumor microenvironment offers an explanation to the final effect of a triggered immune response with an effective response or an immunosuppressive effect resulting in tolerance-anergy [10].

## 3. The Immune Synapses: Role of the Antigen 4 Associated to Cytotoxic T Lymphocytes (CTLA-4)

**Figure 1:**
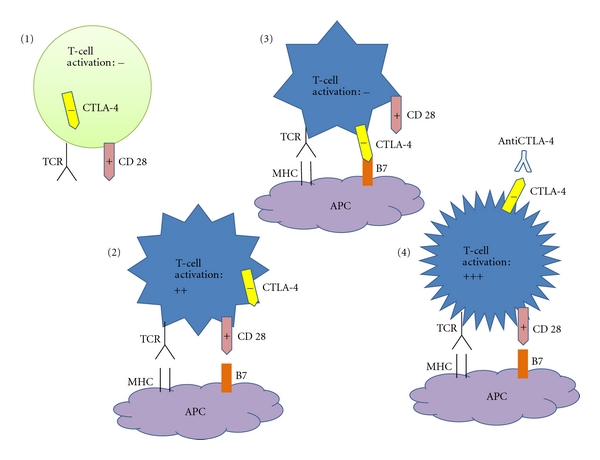
(1) CTLA-4 is a negative regulator of T-cell activation. (2) Conventional T-cells are activated by engagement of MHC and B7. (3) Upon activation, T-cells express CTLA-4 on the cells surface and the union of CTLA-4 with B7 inhibits T-cell activation. (4) Antibody blockade of CTLA-4 produces the liberation of CD28 which could engage with B7 with the best activation of T-cells.

The immune system is a homeostatic system with self-regulating mechanisms that prevent excessive and harmful responses towards the organism that lead to the destruction of normal and healthy cells [1]. One of the key control points in this immune response relies in the HLA-antigen complex recognition by T-cell receptors. This interaction is very complex and involves a series of ligands, such as CD40, a surface molecule that appears early in activated T-cells [7]. This ligand is essential in the generation of antibodies by T-cell induced B lymphocytes as well as in the activation of antigen presenting cells (APCs) which trigger cellular immune responses. The interaction between CD40 ligand and receptor on B-cells and APC upregulates the expression of two surface proteins, CD80 and CD86. When these interact with CD28 on T-cells (immune synapses), T-cells are activated [7]. However, interactions with antigen 4 associated to cytotoxic T lymphocytes (CTLA-4) on T-cells lead to a status of anergy or immune tolerance. Once CTLA-4 (CD152) is generated, immune synapses are mobilized 2-3 days after T-cells are activated, binding to T-cell receptors (TCRs) CD80 and CD86 [11] (Figure 1). CD80 and CD86 preferentially bind to CTLA-4, leading to a decrease in IL-2 production, thus, in activated T-cells. A temporary delay in CLTA-4 appearance on T-cell surface in the immune synapses may trigger RCT and CD28-induced LT activation and expansion, enhancing the immune response [7, 12].

The blockade of CTLA-4 interaction with its ligands can result in an augmentation of antigen specific T-cell responses [13], and several studies have demonstrated that CTLA-4 blockade can enhance immunity to tumors [14, 15]. It has been reported that antibodies against CTLA-4 (anti-CTLA-4) induce proliferation of TCR-stimulated T effector cells and abrogate Treg suppressive activity by enhancing IL-2 and IFN*γ* release in response to polyclonal or tumor antigen stimulation [16]. Curiously, anti-CTLA-4 does not reduce the amount of Tregs, what suggests that anti-CTLA-4 mediates immune responses by direct activation of T effector cells and not by depleting Tregs [16].

There exist 2 CTLA-4 blocking antibodies for use in humans that have been most widely tested in patients with metastatic melanoma [17]. Recently, Ipilimumab has gained FDA approval for clinical use in metastatic melanoma patients after demonstrating benefits in overall survival [18]. Clinical research of anti-CTLA-4 in other solid neoplasms is scarse until now. However, a better understanding of the mechanism of action of anti-CTLA-4, along with its use in the context of combinatorial strategies, may enable to explore the eventual efficacy of these molecules in nonmelanoma tumors, including colorectal cancer [19].

## 4. Prognostic Value of Tumor-Infiltrating Lymphocytes (TILs) and Their Subtypes in Colorectal Cancer

Microscopically, lymphocytes are observed as small cells responding to classical hematoxylin-eosin stains and clearly different from other white cells such as plasmatic cells, neutrophils, eosinophils, macrophages, and masT-cells. In a study published in 1987 by Jass et al. [20], they reported the possibility that lymphocytes infiltrate of the invasive margins of rectal cancer could be an independent prognostic factor for survival, advocating for a new prognostic tool to calculate the risk of this disease. Ropponen et al. [21] confirmed the prognostic value of TILs in colorectal cancer, quantifying them in the tumor stroma and along the invasive margins of the tumor. They subdivided them into four groups according to their histological grade and proved that TILs infiltration was a predictive factor for disease-free and overall survival. An inverse correlation was also observed between the presence of TIL and tumor stage; thus in advanced stages of the disease (Dukes stages C and D), TILs were less numerous than in early stages (Dukes stages A and B) [21].

Follicular and paracortical hyperplasia in local lymphatic nodes are also an important prognostic factor in colorectal cancer. Phil et al. [22] proved in their study that immune response observed in local lymphatic tissue might exert an influence on survival. This study is particularly important as it establishes a correlation between the immune response observed in the tumor layer and in the first lymphatic settlement. Both immune responses are directly related; hence immature dendritic cells migrate from the primary tumor location to the local lymphatic node for maturation and conversion to T-cell antigen presenting cells [22].

In most colorectal tumors, tumoral tissue is infiltrated by a scarce number of lymphocytes and only along the margins of the tumor the highest density of lymphocytes and other inflammatory cells is observed. Proinflammatory cells such as neutrophils and macrophages usually appear with lymphocytes. The latter are usually CD4+ or CD8+ T-cells while B-cells are generally observed in lymphoid follicles [1].

The specific TILs composition has a crucial role in clinical evolution of colorectal cancer. Many research groups have focused their effort on analyzing the eventual relation between T effector cells and regulatory T-cells infiltrates and clinical outcomes. Intraepithelial lymphocytes are mainly CD8 and their number is consistently correlated with higher disease-free survival rates, as proved in several studies [4, 23]. On the contrary, studies that analyze Tregs infiltration report conflicting results [24].

### 4.1. Regulatory T-cells

Treg population represents roughly the 10% of CD4 T-cells and specifically expresses the forkhead box P3 transcription factor (FOXP3) [25, 26] which confers them suppressive properties upon effector T-cells [27, 28]. Increased numbers of FOXP3-infiltrating tumor cell nests have been demonstrated in several neoplasms, and this event is generally associated with unfavourable clinical outcomes. However, there are tumors where Treg infiltration seems to play a different role with protective antineoplastic effects. This is the case of some lymphoproliferative syndromes, especially Hodgkin's disease and follicular lymphoma [29], and probably (but less clear) in colorectal and head and neck carcinomas [30]. Regarding colorectal cancer, Salama et al. [31] after analyzing 967 surgical specimens detected that a high density of Tregs in tumor tissue was associated with better survival, being the only immune biomarker independently associated with overall survival in the multivariate analysis. In the same way, Correale et al. [32] reported a better outcome in advanced colorectal cancer treated with chemo or chemo-immunotherapy if previously there was an intense Tregs infiltration in primary tumors. Two other recent and large studies reported similar results, with favourable prognosis in populations with high FOXP3 T-cell infiltration, at least in the univariate analysis [33, 34].

Ladoire et al. revised in depth this issue and pretended to give a plausible biological explanation based on the different effects of Tregs populations, depending on the diverse and specific microenvironment composition of the tumors [30]. In this sense, they underscore that colorectal carcinomas grow in a “septic microenvironment” where many gastrointestinal bacteria reside and can be translocated across the mucosal surface, inducing proinflammatory and proangiogenic effects, that favour the tumoral growth. In this context, Tregs may suppress the immune reaction induced by these microorganisms and thus counteract their protumorigenic effects. This is an interesting and attractive hypothesis which may explain the improved outcomes associated with Treg infiltration in some neoplastic diseases (hematologic and solid tumors) that have a tight relation with infectious processes.

Although most of the studies advocate for the beneficial effects of Treg infiltration in colorectal cancer (Table 1), there exist other works that could not fully confirmed these results. Sinicrope [35] reported no significant relation between Tregs and prognosis and observed that a low epithelial CD3+/Tregs ratio was associated with shorter disease-free survival. In addition, Camus et al. [36] did not find Tregs infiltration as a reliable marker of good prognosis. Therefore, to date there exist some conflicting results regarding clinical results and accumulation of FOXP3 Tregs in specimens of colorectal cancer and more data are needed to definitely elucidate and establish their role in this disease.

### 4.2. Cytotoxic T CD8+ Cells

In relation to Tregs, results regarding CD8+ infiltration in colorectal cancer are more robust and concordant suggesting strong antitumoral effects and a positive effect on patient survival [24] (Table 2). Diederichsen et al. [37] showed throughout flow cytometry that a low CD4/CD8 ratio is an independent prognostic factor for a better survival. The immunosuppressive role of CD4+, CD25+, and FOXP3+ regulatory T-cells is also elucidated [37].

In 2006, Galon et al. published in Science [39] a very relevant study with clinical-pathological transcendence. Genomic analyses were conducted on 75 cases of colorectal carcinoma in stages I to III and 415 cases with tissue microarrays, observing that tumors with lower rates of recurrence had higher density of immune cells (TCD3, TCD8, memory-TCD45RO, and granzyme B) in the analyzed regions in comparison to recurrent tumors. This study shows that adaptive immunity, expressed by Th1, is inversely proportional to tumor recurrence; thus patients with increased Th1 gene expression present a better prognosis. Furthermore, the centre and margins of the tumor were analyzed finding that, in patients without recurrence, immune cell density was higher in both areas. In patients with low density of total lymphocytes TCD3 and memory lymphocytes (CD45RO+) presented a worse prognosis, similar to those with distant metastasis (stage IV). Patients were stratified according to the UICC-TNM classification, observing that an intense immune response in situ was related to a favourable prognosis despite local extension of the tumor and nodal locoregional infiltration [39]. The authors finally advocate for a redefinition of the diagnostic and histopathological approaches of these tumors as long as immune cell type, density, and location in colorectal carcinoma proved to be a superior prognostic factor and independent from classical prognostic factors in this neoplasia (stage according to the UICC-TNM classification and nodal infiltration). However it is important to notice that it is not possible to absolutely discard an unbalanced selection of the cases due to a higher number of tumors carrying DNA microsatellite instability (MSI) in this study.

Multiple analyses clearly point out that the impact on survival of CD8+ lymphocytes in colon cancer is more obvious with longer follow-up periods [42]. Moreover, in follow-up studies conducted on patients with high or low levels of CD8, survival curves during the first two years are very similar, further separating [24]. Chiba et al. [38] proposed the hypothesis that the presence of CD8+ T-cells in tumor tissue could trigger an immunosurveillance status in the organism, avoiding the development of distant metastasis. Pagès et al. [40] proved that early metastasis development was associated with a poor immune response in tumor tissue. This group demonstrated in 490 patients of colorectal cancer that those patients with a high density of CD45RO+ cells had better prognosis in terms of disease free and overall survival compared with patients with a low density of these memory cells. Tumors without signs of early metastatic invasion had increased infiltrates of immune cells, particularly CD8+ T-cells [40]. Furthermore, Pagès et al. [43] reported in 2009 another study in which they classified 602 early-stage colorectal cancers (stage I and II) into different prognostic groups depending on the density of CD45RO+ and CD8+ cells in two tumor regions (center and invasive margin). Immune classification was found to be an independent prognostic factor in multivariate analysis (*P* < 0.0001), revealing recurrence rates of 4,8% versus 75% in high versus low CD8+ and CD45RO+ infiltration, respectively [43]. Similarly, Mlecnik et al. [44] studied the intratumoral immune infiltrates in a broader population of stage I to IV colorectal cancers, measuring again the lymphocyte infiltrates in the center and the invasive margin of 599 specimens. They used the same immune score of their previous study, defining five patient groups (Im0, Im1, Im2, Im3, Im4). Patients with low densities of CD45RO and CD8 in both tumor regions were classified Im0, and the rest of groups were classified depending upon the density in every tumor region up to the group of four high densities (Im4). In this population, disease free survival and overall survival was far better in the Im3 and Im4 groups, and multivariate analysis confirmed the advantage of the immune score (HR 0,64; *P* < 0,001) compared with the classical TNM staging [44].

### 4.3. DNA Microsatellite Instability

Another issue worthy of consideration is the well-recognized better prognosis of patients with colorectal cancer in the context of Lynch's syndrome [41]. In this sense, DNA microsatellite instability is frequently observed in these hereditary nonpolyposic colorectal cancers and by contrast is relatively uncommon in sporadic colorectal tumors. Usually, tumor epithelium in cases with microsatellite instability is infiltrated by CD3+ and CD8+ lymphocytes, probably resulting from an increased immunologic reconnaissance of mutated proteins on the epithelial surface [45]. Several studies have revealed that microsatellite instability can be associated with a greater T-cell infiltration in tumor tissue [41, 45–47], and hence there has been postulated the hypothesis that this fact might be on the basis of the better clinical outcomes associated with this subgroup of hereditary colorectal cancers. Although this is a plausible explanation, other further prospective studies focusing on histopathologic findings in patients with hereditary nonpoliposic colorectal carcinomas might clarify this question.

### 4.4. Antigen Presenting Cells (APCs)

Along with TILs, antigen presenting cells (APCs) are another components of adaptive immune system worthy of consideration, and among them dendritic cells (DC) are retained as the most potent antigen presenting cells. At present there are numerous studies investigating their role in order to use them in active immunotherapy (vaccines). In colorectal cancer, dendritic cells are found along the invasive margins of the tumor once they have developed completely in lymphoid follicles [48]. The prognostic value of these cells is very important. Dadabayev et al. [49] published that HLA-II cells are distributed in the tumor stroma and that in cases with high density of HLA-II cells, survival was lower; this may be due to the fact that HLA-II cells in those cases are immature as mature cells are scarce in tumor regions. Moreover, overexpressed intercellular adhesion molecule ICAM-1 in tumor stroma fibroblasts could interfere in dendritic cell functions [50]. It is important to remind that tumor reactive T-cells are often anergic because of inappropriate antigen exposure or owed to self recognition; so DCs concourse seems essential to trigger immune-mediated antitumor responses with the ability to generate effector and memory T-cells.

## 5. Immune Effects of Chemotherapy in Colorectal Carcinoma

Colorectal cancer represents a wide group of heterogenic diseases with different clinical behaviours and response to antineoplastic treatments. Nowadays, the main option in advanced disease remains chemotherapy or biochemotherapy. Recently, several studies have revealed that these treatments seem to have a relevant impact on the surrounding stroma and microenvironment [51]. Different cytotoxic drugs destroy tumor cells inducing a type of immunogenic apoptosis, a process of cell death characterized by the activation of caspases and exposure of phosphatidilserine residues in the outer leaflet of the cell [52], and recent studies suggest that this kind of tumoral destruction may improve cancer cell recognition by the immune system [53, 54].

Apoptosis or programmed cell death has been traditionally considered as immunologically “bland” or non-immunogenic. However, this theoretical assumption has not been confirmed in basic and translational research. Rather, it seems that apoptosis is a heterogeneous process that under some circumstances may lead to immunogenic effects [55–57], and this finding is critical to understand better the antineoplastic mechanism of action of some, if not most, chemotherapies.

Oxaliplatin is one of the drugs of choice in advanced colorectal cancer and is included in most of the first line chemotherapy schedules. The group of L. Zitvogel at the Institut Gustave Roussy have studied extensively the immunogenic death of cancer cells induced by chemotherapy, and with respect to oxaliplatin they have demonstrated that it may promote apoptosis in cancer cells via immunogenic effects through two main mechanisms [58, 59].

1. *Early Apoptotic Phase: Calreticulin* (*CRT*). Oxaliplatin induces translocation of the intracytoplasmic protein calreticulin to the cell surface, inducing the apoptotic cell antigen presentation to dendritic cells and stimulating specific antitumor T-cell responses [58, 59].
2. *Late Apoptotic Phase: High Mobility Group Box 1 *(*HMGB1*). Another immunogenic determinant of cell death is the proinflammatory factor HMGB1. HMGB1 is a nuclear protein that is released after necrotic cell death and, as recently reported, from dying cells during late stage apoptosis. After death cell induced by oxaliplatin, HMGB1 may be released in the stroma and act as a neoantigen representing an immunogenic endogenous “danger signal”, and thus initiating an inflammatory response through binding Toll-Like Receptor 4 (TLR4) on DC [59].

**Figure 2:**
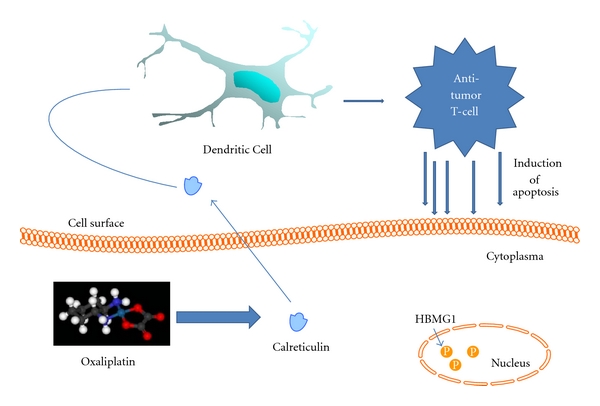
Early Apoptotic Phase: Calreticulin (CRT). oxaliplatin induces translocation of the intracytoplasmic protein calreticulin to the cell surface, inducing the apoptotic cell antigen presentation to dendritic cells and stimulating specific antitumor T-cell responses.

Therefore, immunogenic tumor cell death mediated by chemotherapeutics like oxaliplatin is a multistep process characterized by a temporal sequence of events (Figure 2) including early translocation of calreticulin to the cell surface, and thereafter interaction of CRT with multiple receptors on DC with apoptotic bodies phagocytosis, release and exposure of heat shock proteins, and late release of HMGB1 (60). HMGB1 is able to bind to the TLR4 receptor on DC, which allows tumor-derived antigens to be processed and presented along with MHC and costimulatory molecules on the surface of DC [53, 60]. These mechanisms altogether serve to trigger DC-mediated specific antitumor response, which may be enhanced by the use of costimulatory molecules like GM-CSF or interleukins [7, 61].

Therefore, in contrast with the previous theoretical assumptions, chemotherapeutics like oxaliplatin can induce a highly potent immune response by increasing neoantigen threshold and presentation via antigen presenting cells, with enhancement of T-cell response and generation of memory T-cells [55, 57]. This new paradigm may serve to consider chemotherapeutics as less empirical and more specific drugs, and thus it is tempting to speculate that systemic treatments in colorectal cancer might be customized taking into account their potential effects on tumoral microenvironment. In this sense, there is an interesting field of clinical research to discover that may combine classical CT agents with immunogenic effects with boosting cytokines (GM-CSF, IL2) and new immunogenic molecules like monoclonal antibodies anti-CTLA4 and CD40 agonists. These combinatorial strategies may eventually sustain immunogenic effect of tumoral cell death, enhancing antigen recognition and thus increasing the effector and memory cells specific activity. Regarding this, biomarkers of immune activity should be of the greatest interest, in order to serve as proof of principle of efficacy with an earlier detection of the eventual benefits of oncological treatments in patients. In this sense, changes detected during CT treatments in blood samples, especially in immunophenotype, Tregs amount, and TCD8/Tregs ratio, may represent interesting biomarkers to analyze and validate in the future.

## 6. Conclusions

Scientific evidence supporting the importance of the immune response in neoplastic diseases is growing. In colorectal carcinoma, many studies endorse the prognostic value of TILs infiltration density, depending on the specific subtype of lymphocytes present. Thus, higher densities of effector TCD8 and NK cells in tumor islets and peritumoral tissue seem to be associated with better long-term survival rates.

Despite active research in this field is ongoing and there remain many issues still unsolved, available data support the realization of a systematic histopathological study of the tumor microenvironment along with the classical pathological studies in colorectal cancer. In addition, immune microenvironment may represent a new oncological target from a therapeutic perspective, giving rise to a new promising chance of clinical research to our patients.

## Figures and Tables

**Table 1:** Studies of tumor-infiltrating immune cells (Tregs) and prognosis in colorectal carcinoma.

Study	*n*	Immune cells	Findings: correlation with prognosis
Salama et al. [31]	967	CD8+, CD45RO+, and FOXP3+ tumor-infiltrating	Positive correlation for T-regs in tumor, negative in normal mucosa
Correale et al. [32]	57	CD4+, CD8+, and FOXP3+ T-cells in stroma adjacent to neoplastic glands	Positive correlation for T-regs
Sinicrope et al. [35]	160	CD4+, CD8+, CD25+, and FOXP3+ T-cells	Negative correlation for T-regs. Positive correlation for CD3+ T-cells
Frey et al. [33]	1420	FOXP3+ T-cells	Positive correlation for FOXP3+ T-cells
Nosho et al. [34]	768	CD3+, CD8+, CD45RO+, and FOXP3+ T-cells	Positive correlation for CD8+, CD45RO+, and FOXP3+ T-cells.

**Table 2:** Studies of tumor-infiltrating immune cells (Cytotoxic T CD8+ cells and CD45RO+) and prognosis in colorectal carcinoma.

Study	*n*	Immune cells	Findings: correlation with prognosis
Naito et al. [4]	131	CD8+ and GrB+ tumor-infiltrating cells.	Positive correlation for CD8+ T-cells
Jass et al. [20]	104	Tumor-infiltrating S-100+, HLA class II+, CD208+, CD1a+ dendritic cells.	Negative correlation for dendritic cells
Chiba et al. [38]	371	CD8+ T-cells within cancer cell nests	Positive correlation for CD8+ T-cells
Galon et al. [39]	490	CD3+, CD8+, GrB+, and CD45RO+ lymphoid infiltrates in tumors/invasive margin	Positive correlation for CD8+ and CD45RO+ T-cell
Pagès et al. [40]	490	CD3+, CD8+, GrB+, and CD45RO+ lymphoid infiltrates in tumors/invasive margin	Positive correlation for CD45RO+ T-cells
Camus et al. [36]	142	CD3+, CD5+, CD8+, CCR+, CD1a+, Ki67+, CD68+, FOXP3+, and cytoDEATH+ tumor-infiltrating cells	Positive correlation for CD8+ and CD45RO+ T-cells
Guidoboni et al. [41]	109	CD3+, CD8+, and GrB+ tumor-infiltrating cells	Positive correlation for CD8+ T-cells
Menon et al. [23]	93	CD4+, CD8+, CD56+, and CD57+ intraepithelial cells.	Positive correlation for CD8+ and CD57+ cells
Diederichsen et al. [37]	41	CD3+, CD8+, and CD4+ tumor-infiltrating cells	Positive for CD8+ T-cells, negative for CD4+ T-cells
Ogino et al. [42]	843	Lymphocytes on top of tumor cells	Positive correlation for lymphocytes
Ropponen et al. [21]	276	Lymphocytic infiltration in the center and periphery of tumors	Positive correlation for lymphocytes
